# Evaluation of VIIRS Land Aerosol Model Selection with AERONET Measurements

**DOI:** 10.3390/ijerph14091016

**Published:** 2017-09-05

**Authors:** Wei Wang, Zengxin Pan, Feiyue Mao, Wei Gong, Longjiao Shen

**Affiliations:** 1 State Key Laboratory of Information Engineering in Surveying, Mapping and Remote Sensing (LIESMARS), Wuhan University, Wuhan 430079, China; wangweicn@whu.edu.cn (W.W.); pzx@whu.edu.cn (Z.P.); weigong@whu.edu.cn (W.G.); 2 School of Remote Sensing and Information Engineering, Wuhan University, Wuhan 430079, China; 3 Collaborative Innovation Center for Geospatial Technology, Wuhan 430079, China; 4 Wuhan Environmental Monitoring Center, Wuhan 430015, China; esabelle520@163.com

**Keywords:** aerosol model, VIIRS, AOD, AERONET

## Abstract

The Visible Infrared Imaging Radiometer Suite (VIIRS) is a next-generation polar-orbiting operational environmental sensor with a capability for global aerosol observations. Identifying land aerosol types is important because aerosol types are a basic input in retrieving aerosol optical properties for VIIRS. The VIIRS algorithm can automatically select the optimal land aerosol model by minimizing the residual between the derived and expected spectral surface reflectance. In this study, these selected VIIRS aerosol types are evaluated using collocated aerosol types obtained from the Aerosol Robotic Network (AERONET) level 1.5 from 23 January 2013 to 28 February 2017. The spatial distribution of VIIRS aerosol types and the aerosol optical depth bias (VIIRS minus AERONET) demonstrate that misidentifying VIIRS aerosol types may lead to VIIRS retrieval being overestimated over the Eastern United States and the developed regions of East Asia, as well as underestimated over Southern Africa, India, and Northeastern China. Approximately 22.33% of VIIRS aerosol types are coincident with that of AERONET. The agreements between VIIRS and AERONET for fine non-absorbing and absorbing aerosol types are approximately 36% and 57%, respectively. However, the agreement between VIIRS and AERONET is extremely low (only 3.51%). The low agreement for coarse absorbing dust may contribute to the poor performance of VIIRS retrieval under the aerosol model (*R* = 0.61). Results also show that an appropriate aerosol model can improve the retrieval performance of VIIRS over land, particularly for dust type (*R* increases from 0.61 to 0.72).

## 1. Introduction

Atmospheric aerosols significantly influence the radiation budget of the Earth by affecting precipitation rates, the lifetime and microphysical properties of clouds, and tropospheric photochemistry [1,2,3]. Satellite remote sensing has long been recognized as an ideal approach for monitoring the spatiotemporal distribution of aerosol optical depth (AOD) at the regional and global scales [4]. Aerosol retrieval algorithms have been developed for the global distribution of AOD using different satellite sensors [5,6,7]. The Visible Infrared Imaging Radiometer Suite (VIIRS), which was launched aboard the Suomi National Polar-orbiting Partnership (NPP) Satellite in October 2011, can be used to measure cloud and aerosol properties, ocean color, sea and land surface temperatures, ice motion and temperature, fires, and Earth’s albedo [8,9]. VIIRS is designed with many characteristics similar to the Moderate Resolution Imaging Spectroradiometer (MODIS) [10,11]. VIIRS can obtain aerosol information with finer spatial resolution compared with MODIS, and the obtained information is complementary to the ground measurements from the Aerosol Robotic Network (AERONET) [12,13].

The VIIRS team continuously monitors, evaluates, and improves aerosol retrieval performance [8,14]. Other researchers have evaluated the performance of VIIRS AOD retrievals across different regions [9,15,16,17]. For example, VIIRS AOD was evaluated with ground-measured AOD from over 12 selected AERONET sites and compared with MODIS aerosol data over China in 2013 [15]. The spatiotemporal variations in AOD retrieved from VIIRS in Eastern China were also investigated [16]. Emerging aerosol products from VIIRS, MODIS (Collection 6), and Geostationary Ocean Color Imager in East Asia were evaluated in 2012 and 2013 using ground AOD observations from AERONET and handheld sun photometers [17]. These early validation studies reported that VIIRS data exhibit differences with ground measurements. Therefore, the potential source of uncertainty in VIIRS land aerosol retrievals should be determined to improve VIIRS performance.

Identification of aerosol types is important for the accurate retrieval of AOD from VIIRS [14,18]. When an aerosol type is selected, its optical properties, in terms of refractive indices and size parameters, can be precisely identified and the uncertainty in aerosol retrieval can be effectively reduced [18]. The aerosol model selection algorithm used for VIIRS retrieval is described in detail in [18]; this algorithm can dynamically select the optimal land aerosol model by minimizing the residual between the derived and expected spectral surface reflectance. The standard deviation of the predefined band ratios ranges from 10% to 30%, which indicates that the relationship of the expected spectral surface reflectance obtained from global empirical values is a major error source in VIIRS land aerosol retrieval [18]. Therefore, the dynamic selection of land aerosol models may result in a considerable uncertainty in the land AOD retrieval of VIIRS [14].

Huang, et al. [14] showed the biases and uncertainties of VIIRS AOD under different aerosol models but did not present results to quantify the selection performance of VIIRS aerosol type. Therefore, the present study aims to evaluate the selection performance of a VIIRS aerosol model by comparing aerosol models derived from VIIRS with aerosol types obtained via AERONET. The evaluations and results of this study are potentially useful for optimizing the selection method for an optimal land aerosol model and improving the accuracy of AOD retrieval.

## 2. Datasets and Methods

### 2.1. Datasets

VIIRS is one of the key environmental remote sensing instruments onboard the Suomi NPP satellite. This instrument is a scanning radiometer that can extend and improve the heritage of Advanced Very High Resolution Radiometer (AVHRR) and MODIS [8]. The Intermediate Product (IP) of VIIRS is aerosol retrieval at the pixel level with a high spatial resolution (750 m) [18]. IP is averaged and aggregated to an Environmental Data Record (EDR) with a spatial resolution of 6 km (8 × 8 pixels) [8]. The AOD at 550 nm (AOD550) is identified as good quality at the IP level if the following requirements are satisfied: (1) the solar zenith angle is less than 65°; (2) AOD550 is within 0 to 2; (3) no cloud shadow, adjacent cloud, cirrus, or fire is detected; (4) the surface is not dominated by soil; and (5) the minimum retrieval residual is less than 0.05. The high-quality EDR AOD (8 × 8 pixels) is averaged from good quality IP AODs if the number of good quality pixels is larger than 16. The evaluation in this study only focuses on high-quality VIIRS AOD.

The selected aerosol model is given at the EDR level, which is provided in the VIIRS Aerosol Products Algorithm Theoretical Basis Document [19]. Five predefined aerosol models are found in the VIIRS aerosol algorithm over land: Dust, Smoke High Absorption, Smoke Low Absorption, Urban Clean, and Urban Polluted [18]. These models roughly represent all aerosol types based on AERONET retrievals [20]. They can also be used to retrieve aerosol information from other satellites, such as MODIS and Cloud-Aerosol Lidar and Infrared Pathfinder Satellite Observations (CALIPSO) [10,21]. The aerosol size distributions and the optical characteristics of a given model vary with AOD magnitude [22].

AERONET is a global network of ground-based sun photometers (Cimel sun/sky radiometer) that provide regular and accurate measurements for aerosol optical properties with high spectral and temporal resolutions at different global sites [12]. The total uncertainty of the AERONET level 1.5 AODs is approximately 0.01 to 0.02, which is sufficient to serve as a ground truth value for VIIRS AOD550 validation over land [12,13]. The AERONET sites provided AODs at 340, 380, 440, 500, 675, 870, and 1020 nm and angular distributions of sky radiance at 440, 675, 870, and 1020 nm. Other aerosol optical properties were also provided, such as the single-scattering albedo (SSA) with a 15 min time resolution during daytime. The established second-order polynomial relation between AERONET AODs and wavelengths (340, 380, 440, 500, 675, 870, and 1020 nm) in logarithmic coordinates is applied to calculate AOD550 because AERONET does not provide AOD at the 550 nm channel [14].

### 2.2. Methods

This study investigates the selection of VIIRS aerosol types at the EDR level and compares it with those derived from AERONET level 1.5 data, which cover the period from 23 January 2013 to 28 February 2017. The VIIRS aerosol types were previously described in the literature [18,20]. AERONET retrievals can be classified into four aerosol models, namely, Dust, Mixture, non-absorbing (NA), and black carbon (BC), based on real-time SSA at 440 nm and fine-mode fraction (FMF) at 550 nm, of which the classification criteria (Table 1) for AERONET aerosol can be found in the study of Lee, et al. [23]. FMF was applied to represent the dominant mode for size distribution, and SSA was applied to separate aerosols with different absorption levels [23].

The advantages of this algorithm are its simplicity and robustness, but performance depends on threshold values. O’Neill, et al. [24] showed that the AERONET retrieval algorithm tends to overestimate fine-mode AOD and underestimate coarse-mode AOD because it used a threshold of 0.6 mm to distinguish between fine- and coarse-mode aerosols. Consequently, by adopting a safety margin of 0.2, fine-mode aerosols are defined by FMF to be greater than 0.6, whereas coarse-mode aerosols are defined to be less than 0.4 [23]. Dubovik, et al. [20] found that SSAs at 440 nm are within 0.90–0.98 for urban/industrial aerosol, 0.89–0.95 for biomass burning, and 0.92–0.93 for desert dust. Biomass burning aerosol contains BC, whereas urban/industrial aerosol contains BC and NA. Thus, the SSA threshold of 0.95, which is the upper limit of SSA for biomass burning aerosol, is used to distinguish between absorbing and non-absorbing aerosols. This threshold is also acceptable for distinguishing between desert dust (SSA range of 0.92–0.93) and oceanic aerosols (SSA of 0.98) in coarse-mode particles.

**Figure 1:**
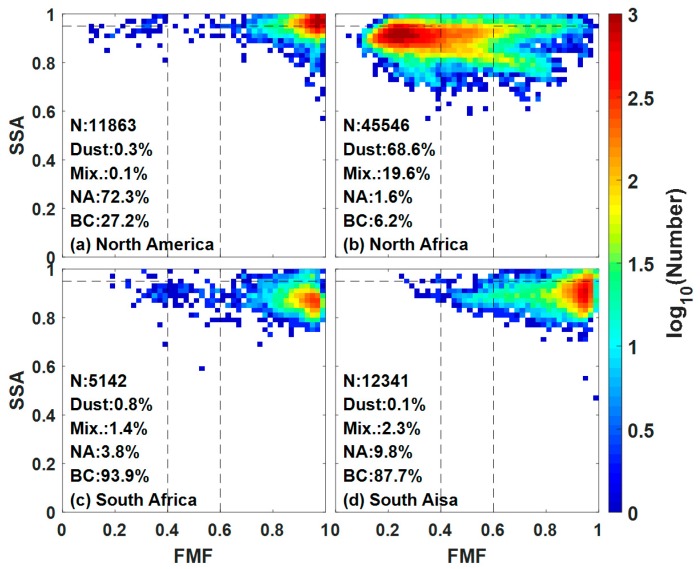
Scatterplots of FMF and SSA in four typical regions: (**a**) North America; (**b**) North Africa; (**c**) South Africa; and (**d**) South Asia. Abbreviation in this figure: Mix. (Mixture), non-absorbing (NA), black carbon (BC), single-scattering albedo (SSA) and fine-mode fraction (FMF).

The present study investigates the performance of the aforementioned method and displays the scatterplots of FMF and SSA in four typical regions shown in Figure 1. North America (30° to 60° N, −135° to −45° E) is affected by aerosols mainly from non-absorbing anthropogenic pollution and wild fires. The most frequently detected aerosol type is NA, followed by BC, whereas mixture and dust are rarely detected in North America. The most frequently detected aerosol type in North Africa (0° to 30° N, −20° to 65° E) is dust from the Sahara Desert. The dominance of BC in South Africa (−40° to 0° N, −20° to 65° E) and South Asia (−10° to 20° N, 65° to 180° E) arises from periodic biomass burning. The remarkable difference between the two regions is evident in the BC content of biomass-burning aerosols. More BC is generated from the flaming stage of burning grass in Africa than from the smoldering stage of burning forests or straw in South Asia [25]. Consequently, the classification criteria can reasonably classify AERONET aerosols into four types.

For comparison, we selected the VIIRS aerosol types and their corresponding AERONET aerosol types based on the VIIRS Aerosol Products Algorithm Theoretical Basis Document [19], as shown in Table 1. No clear criterion distinguishes among ‘Low Absorption Smoke’, ‘High Absorption Smoke’ and ‘Polluted Urban’ because the SSAs of the three aerosol models have overlaps. Thus, we used the BC of AERONET to represent the three VIIRS aerosol types with different absorption levels. The Mixture type of AERONET has no corresponding VIIRS aerosol type.

The following rules based on [8,15,17] were executed to obtain the matchups between the datasets of AERONET and VIIRS. We collected all the VIIRS aerosol types within a 27.5 km radius circle that centered on the AERONET sites. AERONET data were acquired within 30 min of VIIRS satellite overpass times. The threshold values of 27.5 km and 30 min were obtained from the evaluation study of VIIRS retrievals executed by the VIIRS aerosol calibration/validation team [8,14]. Multiple VIIRS aerosol types may exist within the 27.5 km circle (including approximately 80 VIIRS EDR retrievals) at a given time. Thus, we selected the most common aerosol types (over 50% of the total valid number) for each matchup with a well-represented type. To match AOD between VIIRS and AERONET, the VIIRS AODs (within the radius of 27.5 km and time of 30 min) near each AERONET site were averaged to reduce evaluation uncertainty. We removed the matchups without a dominant aerosol type or those where the high-quality VIIRS dataset is less than 20% within the matching range. Consequently, a matchup occurs when there exists a dominant model (greater than 50% of the ~80 EDRs within the circle centered over the AERONET site are the same) in the VIIRS retrieval that exists within 30 min of an AERONET observation. The hit ratio for each aerosol type from all the matchups was calculated to quantify aerosol type agreement between VIIRS and AERONET. The “hit ratio” is the number of “matchups” that agrees with AERONET relative to the total number of “matchups”.

To demonstrate the effects of the aerosol model on VIIRS retrieval, the performance of high-quality VIIRS AOD at 550 nm was evaluated by comparing with the AERONET measurements under each VIIRS aerosol type. The following evaluation methods were applied: (1) accuracy, which refers to the average bias between two datasets; (2) precision, which is the standard deviation of the bias; (3) correlation coefficient (*R*), which indicates the correlation and dependence of statistical relationships between two datasets; (4) linear regression (*AOD_VIIRS_* = *a* × *AOD_AERONET_* + *b*), which is used to estimate the slope (*a*) and intercept (*b*) of the datasets; and (5) the percentage of VIIRS AODs that falls within the MODIS expected error (EE) of ± (0.05 + 0.15 AOD) over land [8,26]. VIIRS is expected to retrieve aerosol properties with similar or even better performance than those of MODIS [8]; thus, the MODIS uncertainty applied in this study can assess whether VIIRS AOD can achieve the accuracy of MODIS.

## 3. Results and Analysis

**Figure 2:**
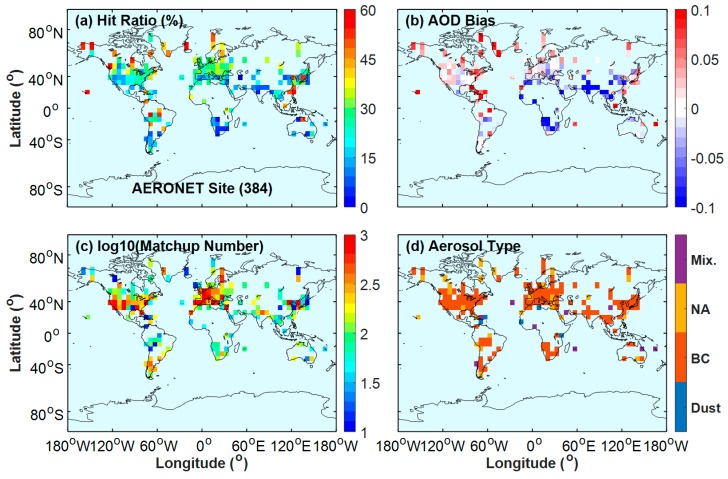
AERONET sites with more than 10 matchups applied in the study. All AERONET sites in a 6° × 6° grid box throughout the world: (**a**) hit ratio of aerosol type between VIIRS and AERONET; (**b**) AOD difference (VIIRS minus AERONET); (**c**) matchup number of VIIRS and AERONET; and (**d**) aerosol type with the highest frequency.

This section compares the aerosol types from VIIRS with those from AERONET. There are 384 AERONET sites were applied in this study. The colored boxes in Figure 2a show the hit ratio of the aerosol type at each 6° × 6° grid box, and each of the 384 sites has more than 10 comparison matchups. The hit ratio indicates the agreement percentage of the aerosol type for each grid box, which is the percentage of the days with coincident aerosol type to the total matchup number at each grid box (Figure 2c). Figure 2b displays the mean AOD difference between the AODs of the VIIRS and AERONET sites.

Figure 2a indicates that the relatively large hit ratios (about 28%) occur in the developed regions of Europe and the Eastern United States, where NA and BC are the most common aerosol types (Figure 2d). The bias between VIIRS and AERONET AODs over these regions can be disregarded (only −0.01 ± 0.03). However, large AOD underestimation (−0.08 ± 0.09), which corresponds to a low hit ratio of approximately 6%, can be observed over South Africa due to the influence of coarse dust aerosols. Similarly, the low hit ratios (approximately 11%) over India and Northern China correspond to a large underestimation (−0.10 ± 0.23). For these developing countries (i.e., India and China), polluted urban aerosol should be dominant due to high polluting industries and fossil fuel consumption. Therefore, the misidentification of the aerosol model (low hit ratio) may be linked to these biases. The influence of aerosol model misidentification on VIIRS aerosol retrieval is further analyzed in the next paragraph.

**Figure 3:**
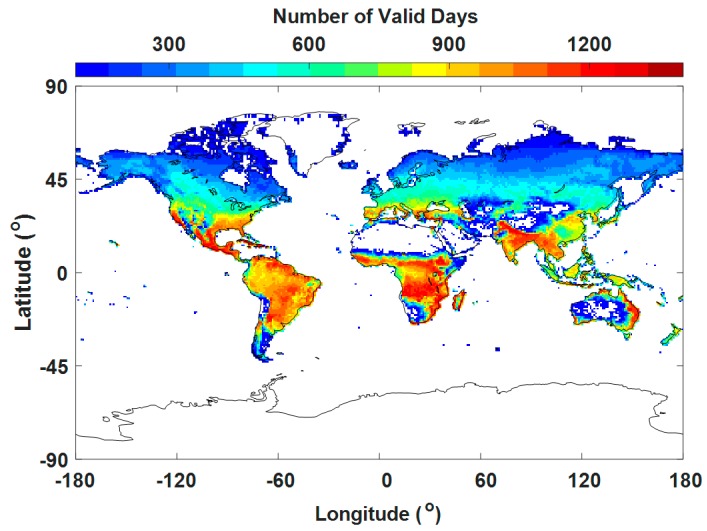
Valid days of VIIRS at a grid of 1° × 1° during the study period (total of 1497 days from 23 January 2013 to 28 February 2017).

**Figure 4:**
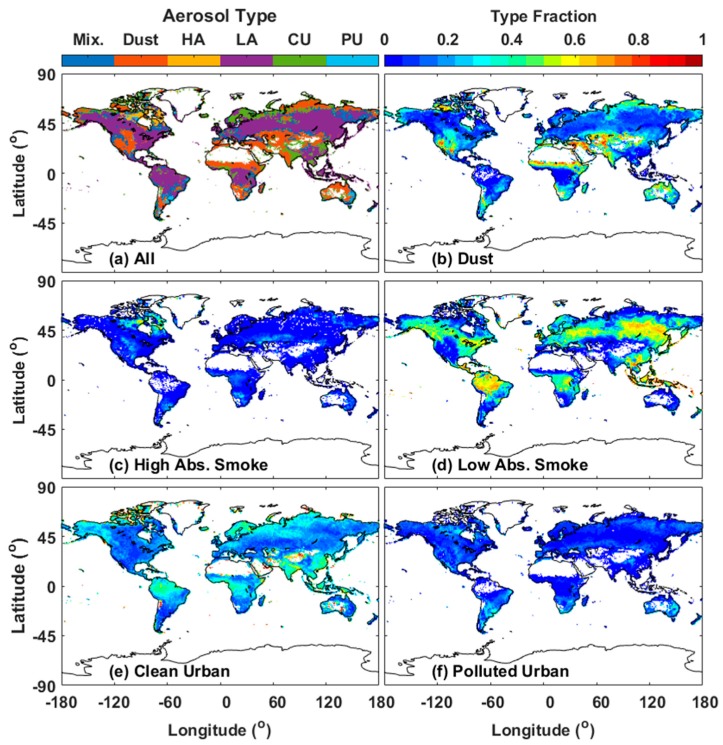
Spatial distribution of aerosol types from VIIRS: (**a**) aerosol types (left color bar) with the highest frequency in each pixel; The fraction (right color bar) for (**b**) Dust; (**c**) Smoke High Absorption (**d**) Smoke Low Absorption (**e**) Clean Urban and (**f**) Polluted Urban of VIIRS aerosol types. Abs. refers to Absorption in each sunfigure.

Figure 3 displays the valid days of VIIRS at a grid of 1° × 1° during the study period (total of 1497 days). The valid days refer to the number of days with high-quality VIIRS retrievals at each 1° × 1° grid from 23 January 2013 to 28 February 2017. The regional distribution of the dominant land aerosol model in each 1° × 1° grid is shown in Figure 4a. The fractions of each aerosol type for VIIRS are displayed in Figure 4b–f. The fraction of an aerosol type is the ratio of the days of the aerosol type to the total valid days at each 1° × 1° grid (Figure 3). The dominant land aerosol model for Figure 4a was obtained from the mode value in Figure 4b–f at each 1° × 1° grid. The void regions in Figure 4 are due to the limitation of VIIRS in retrieving high-quality AOD over an area with scarce vegetation.

VIIRS AODs were overestimated over the Eastern United States and the developed regions of East Asia, as shown in Figure 2b. The clean urban model is appropriate for the Western United States and the developed regions of East Asia (i.e., South Korea, Japan, and Taiwan). However, low-absorption smoke is the most selected model for VIIRS (Figure 4a,e). Low-absorption smoke exhibits stronger absorption than clean urban, which may result in a positive AOD bias. Underestimated AOD displays are observed in Southern Africa, India, and Northeastern China (Figure 2b). The dominant aerosol model for Southern Africa is high-absorption fine particles due to savanna fires. But the local aerosol may mix with coarse dust originated from Northern Sahara. Dust particles with a coarse size exhibits lower scattering capability than fine smoke particles, dust model mixing with fine smoke particles may result in a AOD bias [14]. The developing regions of India and Northeastern China mostly have a Polluted Urban model due to the massive incomplete burning of fossil fuels with low processing technology for their industries and activities. However, the Clean Urban model is selected as the dominant aerosol model for these developing regions as shown in Figure 4a,e. The lower absorption of the Clean Urban model than that of the Polluted Urban model may account for the underestimations. In summary, the misidentification of VIIRS aerosol types may contribute to VIIRS retrieval overestimation over the Eastern United States and the developed regions of East Asia and the underestimation over Southern Africa, India, and Northeastern China.

**Figure 5:**
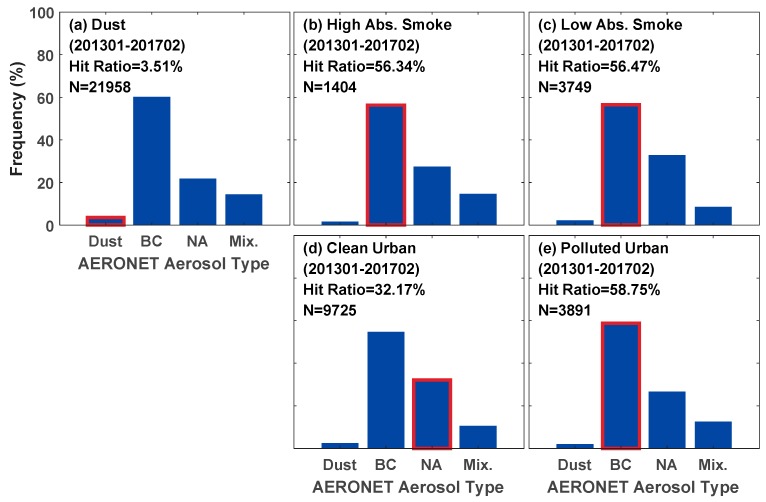
Corresponding AERONET aerosol types for each VIIRS aerosol type. The red box indicates the AERONET aerosol type that is consistent with that of VIIRS. The *y*-axis denotes the frequency of different types of AERONET at a given VIIRS aerosol type: (**a**) Dust; (**b**) High Absorption Smoke; (**c**) Low Absorption Smoke; (**d**) Clean Urban; and (**e**) Polluted Urban.

The hit ratio is defined as the percentage of the number of coincident aerosol types to the number of total matchups (N). The histograms of the comparison of the aerosol types are shown in Figure 5, where the red box around a bar indicates that the VIIRS aerosol type is consistent with the AERONET type. Overall, we found 40,727 matchups for comparison, with an agreement of 22.33% for all the cases. The Dust model is frequently selected (53.92%, 21,958 of the collection number), followed by the Urban Clean (23.88%), Urban Polluted (9.55%), Smoke Low Absorption (9.21%), and Smoke High Absorption (3.45%) models. The VIIRS dust type has the most number of matchups. However, the dust type agreement between VIIRS and AERONET is extremely low (only 3.51%). AERONET BC exhibits the highest frequency for VIIRS dust type. However, the two types have a considerable difference in effective particle radius (BC is a fine absorbing particle, whereas dust is a coarse particle), which may result in a large bias in VIIRS aerosol retrieval under dust type. The agreements range from 32% to 58% for fine aerosol types, i.e., 56.34% for high-absorption smoke, 56.47% for low-absorption smoke, 32.17% for clean urban, and 58.75% for polluted urban.

VIIRS can determine fine dominant aerosols better than coarse absorbing aerosols (Dust). Large amounts of VIIRS dust selection reduce the overall agreement between VIIRS and AERONET aerosol types. Another considerable point is the differences in the aerosol classification criteria between VIIRS and AERONET, as shown in Table 1. For example, Absorption Smoke and Polluted Urban correspond to the same AERONET aerosol type because no appropriate criterion can distinguish between them. Moreover, the inherent subjectivity of the classification criteria may reduce the reliability of the comparison although our threshold values for the classification criteria are coincident with those in [23].

**Figure 6:**
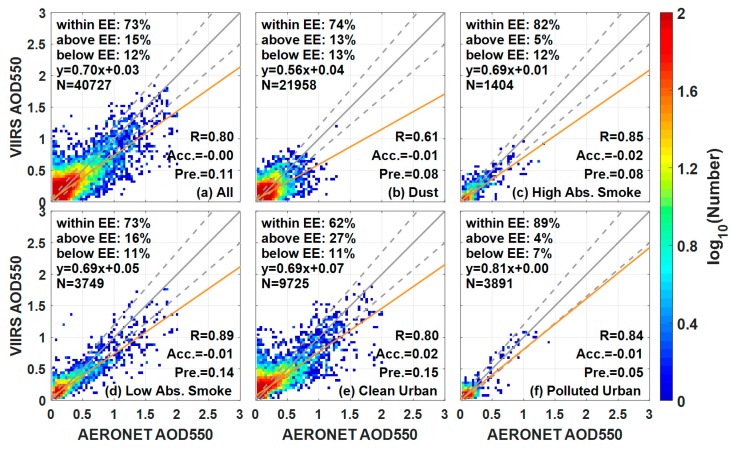
Scatterplots of VIIRS AOD550 against AERONET measurements under different VIIRS aerosol types: (**a**) All; (**b**) dust; (**c**) High Absorption Smoke; (**d**) Low Absorption Smoke; (**e**) Clean Urban and (**f**) Polluted Urban. The width of each pixel is 0.04, and the number of collocations that falls within/above/below EE is represented in each figure. The yellow line is the regression line, the gray solid line is the 1:1 line, and the gray dashed lines are the EE envelopes. Abbreviation in this figure: expected error (EE), accuracy (Acc.) and precision (Pre.).

The comparison between VIIRS EDR AODs and ground AERONET observations (N = 40,727) shows an *R* of 0.80, a linear regression slope of 0.70, a positive intercept of 0.03, and an accuracy equal to 0.00, with 73% of the AODs falling within the EE in Figure 6a. A worldwide evaluation study conducted from 23 January 2013 to 31 December 2014 exhibits similar results with matchup numbers (29,145), accuracy (−0.0008), precision (0.116), uncertainty (0.116), and *R* (0.817) in land AOD versus AERONET [14]. However, Figure 6 shows the varying retrieval errors of VIIRS AOD under different aerosol types. VIIRS AOD products exhibit overestimation (accuracy = 0.02) for Clean Urban but underestimation (accuracy = −0.02 to −0.01) for other aerosol types. The Urban Clean and the Low Absorption Smoke models demonstrate higher uncertainties compared with the other three models, and similar results can be found in [14]. For VIIRS AOD under the Clean Urban model, only 62% of the retrievals fall within EE. For the inter-comparison under different aerosol types, VIIRS exhibits the lowest *R* (0.61) under the coarse Dust model. The *R* values for the other fine aerosol models range from 0.80 to 0.89. The poor performance of VIIRS retrieval under the coarse Dust model may be attributed to the large proportion of misidentification of dust types.

**Figure 7:**
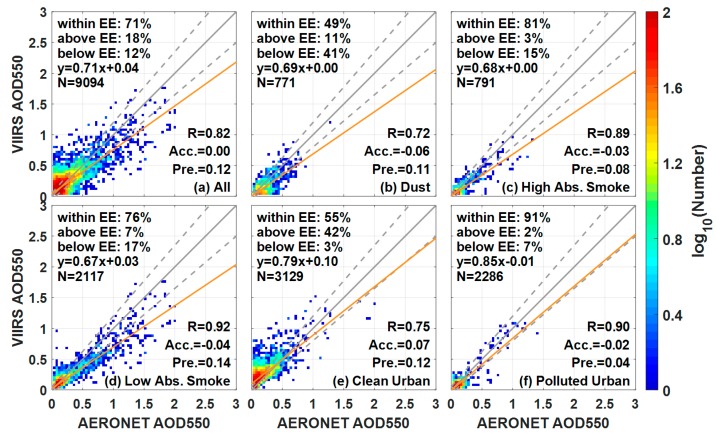
VIIRS aerosol retrievals and AERONET measurements agree on the aerosol type. The scatterplots of the VIIRS AODs against AERONET measurements under different VIIRS aerosol types: (**a**) All; (**b**) dust; (**c**) High Absorption Smoke; (**d**) Low Absorption Smoke; (**e**) Clean Urban and (**f**) Polluted Urban. The width of each pixel is 0.04, and the number of collocations that falls within/above/below EE is represented in each figure. The yellow line is the regression line, the gray solid line is the 1:1 line, and the gray dashed lines are the EE envelopes.

Figure 7 shows the VIIRS performance when VIIRS and AERONET agree on the aerosol type. A slight improvement in VIIRS performance can be observed in fine absorbing aerosols (i.e., the Smoke and Polluted Urban models) while an evident increased correlation (*R* increases from 0.60 to 0.72) is observed in the dust type aerosol. Moreover, the appropriate aerosol model cannot improve VIIRS performance under NA aerosol (clean urban), which may suggest that VIIRS aerosol retrieval is insensitive to this model. Overall, selecting the appropriate aerosol model can improve the retrieval performance of VIIRS, particularly for dust.

## 4. Conclusions

The VIIRS sensor is a next-generation polar-orbiting operational environmental sensor with a capability for global aerosol observations. The identification of land aerosol types is significant to reduce the uncertainty of VIIRS retrieval. This study quantitatively evaluated the performance of VIIRS aerosol type selection by comparing with AERONET observations (level 1.5) from 23 January 2013 to 28 February 2017 and analyzed the influence of a misidentified aerosol model on retrieval. The spatial distribution of VIIRS aerosol types and the AOD bias between VIIRS and AERONET demonstrate that the misidentification of VIIRS aerosol types may contribute to VIIRS retrieval overestimation over the Western United States and the developed regions of East Asia and underestimation over Southern Africa, India, and Northeastern China. Overall, VIIRS aerosol types exhibit an agreement of 22.33% with AERONET for all cases. VIIRS dust type achieves the most number of matchups (53.92%). However, the dust type agreement between VIIRS and AERONET is extremely low (only 3.51%). The agreements for fine non-absorbing and absorbing aerosol types are approximately 36% and 57%, respectively. The low agreement for coarse absorbing dust may contribute to the poor performance of VIIRS retrieval under the aerosol model (*R* = 0.61). An appropriate aerosol model can improve the retrieval performance of VIIRS, particularly for dust type (*R* increases from 0.61 to 0.72). Our results indicate that the automatic selection method for an optimal aerosol model over land should be improved further.

## Figures and Tables

**Table 1:** VIIRS aerosol types and their corresponding AERONET aerosol types. Abbreviation in this table: Aerosol Robotic Network (AERONET), non-absorbing (NA), black carbon (BC), single-scattering albedo (SSA) and fine-mode fraction (FMF).

VIIRS Aerosol Types	Characteristics and Regions	AERONET Aerosol Types	AERONET Classification Criteria
Dust	Absorption and coarse mode, Sahara, Middle East	Dust	FMF < 0.4 and SSA ≤ 0.95
High Absorption Smoke	High absorption, South Africa, savanna fires	BC	FMF > 0.6 and SSA ≤ 0.95
Low Absorption Smoke	Low absorption, South America, woody burning	BC	FMF > 0.6 and SSA ≤ 0.95
Clean Urban	Low absorption, developed regions	NA	FMF > 0.6 and SSA > 0.95
Polluted Urban	High absorption, developing regions	BC	FMF > 0.6 and SSA ≤ 0.95
Not available	Not available	Mixture	0.4 ≤ FMF ≤ 0.6

## References

[1] Twomey S. (1991). Aerosols, clouds and radiation. Atmos. Environ. Part A Gen. Top..

[2] Pan Z., Gong W., Mao F., Li J., Wang W., Li C., Min Q. (2015). Macrophysical and optical properties of clouds over east asia measured by calipso. J. Geophys. Res. Atmos..

[3] Pan Z., Mao F., Gong W., Min Q., Wang W. (2017). The warming of Tibetan Plateau enhanced by 3D variation of low-level clouds during daytime. Remote Sens. Environ..

[4] Wang W., Mao F., Du L., Pan Z., Gong W., Fang S. (2017). Deriving hourly PM_2.5_ concentrations from himawari-8 aods over Beijing-Tianjin-Hebei in China. Remote Sens..

[5] Hauser A., Oesch D., Foppa N., Wunderle S. (2005). Noaa avhrr derived aerosol optical depth over land. J. Geophys. Res. Atmos..

[6] Torres O., Tanskanen A., Veihelmann B., Ahn C., Braak R., Bhartia P.K., Veefkind P., Levelt P. (2007). Aerosols and surface UV products from ozone monitoring instrument observations: An overview. J. Geophys. Res. Atmos..

[7] Sayer A.M., Hsu N.C., Bettenhausen C., Jeong M.J. (2012). Global and regional evaluation of over-land spectral aerosol optical depth retrievals from seawifs. Atmos. Meas. Tech..

[8] Liu H., Remer L.A., Huang J., Huang H.-C., Kondragunta S., Laszlo I., Oo M., Jackson J.M. (2014). Preliminary evaluation of S-NPP viirs aerosol optical thickness. J. Geophys. Res. Atmos..

[9] Wang W., Mao F., Pan Z., Du L., Gong W. (2017). Validation of viirs AOD through a comparison with a sun photometer and modis aods over wuhan. Remote Sens..

[10] Levy R.C., Remer L.A., Mattoo S., Vermote E.F., Kaufman Y.J. (2007). Second-generation operational algorithm: Retrieval of aerosol properties over land from inversion of moderate resolution imaging spectroradiometer spectral reflectance. J. Geophys. Res. Atmos..

[11] Nichol J., Bilal M. (2016). Validation of modis 3 km resolution aerosol optical depth retrievals over asia. Remote Sens..

[12] Holben B., Eck T., Slutsker I., Tanre D., Buis J., Setzer A., Vermote E., Reagan J., Kaufman Y., Nakajima T. (1998). Aeronet—A federated instrument network and data archive for aerosol characterization. Remote Sens. Environ..

[13] Dubovik O., Smirnov A., Holben B.N., King M.D., Kaufman Y.J., Eck T.F., Slutsker I. (2000). Accuracy assessments of aerosol optical properties retrieved from aerosol robotic network (aeronet) sun and sky radiance measurements. J. Geophys. Res. Atmos..

[14] Huang J., Kondragunta S., Laszlo I., Liu H., Remer L.A., Zhang H., Superczynski S., Ciren P., Holben B.N., Petrenko M. (2016). Validation and expected error estimation of Suomi-NPP viirs aerosol optical thickness and angström exponent with aeronet. J. Geophys. Res. Atmos..

[15] Meng F., Cao C., Shao X. (2015). Spatio-temporal variability of Suomi-NPP VIIRS-derived aerosol optical thickness over China in 2013. Remote Sens. Environ..

[16] Meng F., Xin J., Cao C., Shao X., Shan B., Xiao Q. (2016). Seasonal variations in aerosol optical thickness over eastern China determined from viirs data and ground measurements. Int. J. Remote Sens..

[17] Xiao Q., Zhang H., Choi M., Li S., Kondragunta S., Kim J., Holben B., Levy R.C., Liu Y. (2016). Evaluation of viirs, goci, and modis collection 6 aod retrievals against ground sunphotometer observations over east asia. Atmos. Chem. Phys..

[18] Jackson J.M., Liu H., Laszlo I., Kondragunta S., Remer L.A., Huang J., Huang H.-C. (2013). Suomi-npp viirs aerosol algorithms and data products. J. Geophys. Res. Atmos..

[19] ATBD (2015). Viirs Aerosol Optical Thickness and Particle Size Parameter Algorithm Theoretical Basis Document (Revision B): 474-00049. http://npp.gsfc.nasa.gov/sciencedocs/2015-06/474-00049_ATBD-VIIRS-AOT-APSP_C.pdf.

[20] Dubovik O., Holben B., Eck T.F., Smirnov A., Kaufman Y.J., King M.D., Tanré D., Slutsker I. (2002). Variability of absorption and optical properties of key aerosol types observed in worldwide locations. J. Atmos. Sci..

[21] Omar A.H., Winker D.M., Vaughan M.A., Hu Y., Trepte C.R., Ferrare R.A., Lee K.P., Hostetler C.A., Kittaka C., Rogers R.R. (2009). The calipso automated aerosol classification and lidar ratio selection algorithm. J. Atmos. Ocean. Technol..

[22] Remer L.A., Kaufman Y.J. (1998). Dynamic aerosol model: Urban/industrial aerosol. J. Geophys. Res. Atmos..

[23] Lee J., Kim J., Song C.H., Kim S.B., Chun Y., Sohn B.J., Holben B.N. (2010). Characteristics of aerosol types from aeronet sunphotometer measurements. Atmos. Environ..

[24] O’Neill N.T., Eck T.F., Smirnov A., Holben B.N., Thulasiraman S. (2003). Spectral discrimination of coarse and fine mode optical depth. J. Geophys. Res. Atmos..

[25] Kaufman Y.J., Tanré D., Boucher O. (2002). A satellite view of aerosols in the climate system. Nature.

[26] Levy R.C., Mattoo S., Munchak L.A., Remer L.A., Sayer A.M., Patadia F., Hsu N.C. (2013). The collection 6 modis aerosol products over land and ocean. Atmos. Meas. Tech..

